# Predictive and Prognostic Factors in Definition of Risk Groups in Endometrial Carcinoma

**DOI:** 10.5402/2012/325790

**Published:** 2012-11-14

**Authors:** Bengt Sorbe

**Affiliations:** Department of Oncology, Örebro University Hospital, 701 85 Örebro, Sweden

## Abstract

*Background*. The aim was to evaluate predictive and prognostic factors in a large consecutive series of endometrial carcinomas and to discuss pre- and postoperative risk groups based on these factors. *Material and Methods*. In a consecutive series of 4,543 endometrial carcinomas predictive and prognostic factors were analyzed with regard to recurrence rate and survival. The patients were treated with primary surgery and adjuvant radiotherapy. Two preoperative and three postoperative risk groups were defined. DNA ploidy was included in the definitions. Eight predictive or prognostic factors were used in multivariate analyses. *Results*. The overall recurrence rate of the complete series was 11.4%. Median time to relapse was 19.7 months. In a multivariate logistic regression analysis, FIGO grade, myometrial infiltration, and DNA ploidy were independent and statistically predictive factors with regard to recurrence rate. The 5-year overall survival rate was 73%. Tumor stage was the single most important factor with FIGO grade on the second place. DNA ploidy was also a significant prognostic factor. In the preoperative risk group definitions three factors were used: histology, FIGO grade, and DNA ploidy. *Conclusions*. DNA ploidy was an important and significant predictive and prognostic factor and should be used both in preoperative and postoperative risk group definitions.

## 1. Introduction

Endometrial carcinoma is the most common cancer of the female genital tract in the western world. Worldwide 287,000 new cases are diagnosed annually with this disease. Endometrial carcinomas are generally thought to have a favorable prognosis due to early detection, and the majority of tumors are detected in early stages. However, in fact this is not fully true, and there are important subgroups within this diagnosis with poor prognosis and outcome of treatment. Therefore, the first step to improve the situation has been to find predictive and prognostic factors, then to define clinically relevant risk groups, and finally to design clinical trials and treatment options for these risk groups.

Unfortunately, no consensus exists on which predictive or prognostic factors that should be used and how to combine them in the definition of suitable-risk groups. As a result of this, the randomized phase III trials presented during the last decades are difficult to compare since these definitions have varied, more or less, in most of them.

Another problem has been the small size and low power of most studies in the literature dealing with prognostic and predictive factors. Despite more or less sophisticated statistical methods with multivariate technique, the results are not reliable enough for definitive conclusions from such small series analyzing multiple variables. A few exceptions do exist but then with data from large registry studies, but then with other problems of selection and bias built in.

Six prospective randomized studies have been presented since 1980 to elucidate the value of external beam pelvic radiotherapy after surgery in early-stage endometrial carcinoma (Aalders, PORTEC-1, GOG#99, ASTEC/EN.5, PORTEC-2, and Sorbe) [1–7]. The treated populations varied in all these studies from no risk groups defined (Aalders) [1] to a mixture of low-risk (PORTEC-1, GOG#99) [2, 3], medium-risk (PORTEC-1, GOG#99, ASTEC/EN.5, and PORTEC-2) [2–5], or high-risk cases (ASTEC/EN.5) [4]. Type of primary surgery and staging also varied from no staging at all (Aalders, PORTEC-1, ASTEC/EN.5, and PORTEC-2) [1, 2, 4, 5] to staging with lymph node sampling or complete lymphadenectomy (GOG#99, ASTEC/EN.5) [3, 4]. Subgroup analyses performed within the frame of these studies have suffered from low power, and no level one data are presented for well-defined medium-risk or high-risk groups.

Three prospective randomized trials of low-risk, medium-risk, and high-risk cancers have been performed in Sweden and some other European countries. Vaginal brachytherapy, external beam pelvic radiation, and adjuvant chemotherapy were addressed in these studies. These three studies are now published [7–9].

In the present retrospective study a large, comprehensive, and consecutive series of more than 4,500 endometrial carcinomas in FIGO stages I–IV were analyzed with regard to predictive and prognostic factors and definition of the risk groups used in the above mentioned three prospective randomized studies. Special emphasis will be made on the prognostic value of DNA ploidy and the importance of this factor in the risk group definitions.

## 2. Material and Methods 

### 2.1. Patients

One Swedish Cancer Center (Örebro) for gynecological oncology recruited patients with all stages (FIGO I–IV) of endometrial carcinomas in an observation study. The period of recruitment was from January 1975 to December 2009. In all, 4,543 patients were included. Postoperative external pelvic irradiation and/or vaginal brachytherapy were administered to the majority of the patients. No further treatment-related details were analyzed in this study. The median age of the patients was 67 years (range 23–99 years). Tumor characteristics are presented in Tables 1 and 2.

### 2.2. Risk Group Definitions

The definition of high-risk carcinomas was as follows: (1) FIGO stage I, (2) nonendometrioid histological type, (3) presence of two of the following risk factors: FIGO grade 3 (poorly differentiated), deep (≥50%) myometrial infiltration, DNA aneuploidy (FCM), (4) nuclear grade 3, (5) pathologically negative lymph nodes, and (6) negative abdominal cytology. Points 5-6 were optional in this study, and data are not available for all cases. 

The definition of medium-risk carcinomas was as follows: (1) FIGO stage I, (2) endometrioid histological type, (3) presence of one of the following risk factors: FIGO grade 3 (poorly differentiated), deep (≥50%) myometrial infiltration, DNA aneuploidy (FCM), (4) nuclear grade 1-2, (5) pathologically negative lymph nodes, and (6) negative abdominal cytology. Points 5-6 were optional in this study, and data are not available for all cases. Lymph vascular space invasion (LVSI) was not regularly included in the pathology reports at the participating centers and was not included in the definition of the medium-risk group. 

The definition of low-risk carcinomas was as follows: (1) FIGO stage I, (2) endometrioid histological type, (3) presence of none of the following risk factors: FIGO grade 3 (poorly differentiated), deep (≥50%) myometrial infiltration, DNA aneuploidy (FCM), or (4) nuclear grade 3. All pathology reports were reviewed by one experienced pathologist at the regional referral center.

### 2.3. Primary Surgery

The primary surgery was total abdominal hysterectomy, bilateral salpingo-oophorectomy, appendectomy, node sampling of enlarged lymph nodes, and peritoneal washing with cytology. Lymphadenectomy (pelvic ± paraaortic) was not performed as a routine at the centers referring patients to the regional clinic. The surgery was performed at five departments of Gynecology and Obstetrics, but all patients were then referred to a Gynecologic Oncology Department for postoperative evaluation and treatment. The time interval between surgery and brachytherapy ± external pelvic irradiation was 4–8 weeks. All patients were then planned for a 10-year follow-up program. The median follow-up period at the time of analysis was 115 months (range 1–362 months) for patients alive. During all visits, symptoms and signs related to the therapy were recorded, but in this study treatment-related side effects are not presented.

### 2.4. Brachytherapy

For the brachytherapy treatments, MicroSelectron HDR machines with an iridium source (Ir-192) were used. Plastic vaginal cylinders with a diameter of 20 mm, 25 mm, or 30 mm were used as standard. The diameter of the cylinder was individually chosen to ensure good contact between the surface of the applicator and the vaginal mucosa. The length of the vagina was measured from the vault to the level of introitus. The proximal 2/3 of the vaginal length was defined as the target volume. The dose per fraction was specified at a depth of 5 mm from the surface of the vaginal cylinder with the HDR technique. Library dose plans that covered different vaginal lengths in steps of 10 mm and the different diameters of the cylinders were used. The dose calculations were made on the Nucletron Planning System (NPS v. 10) and the PLATO Brachytherapy Planning System (BPS v. 14) at centers using this equipment. Six fractions were given during an 8-day period. The dose per fraction was assigned to 2.5–3.0 Gy. Thus, the total doses delivered were 15.0–18.0 Gy. Recalculated to 2-Gy-equivalent doses (EQD_2_), the total doses were 15.6–19.5 Gy at a depth of 5 mm (*α*/*β* = 10.0). All treatments were given on an outpatient basis.

### 2.5. External Beam Radiotherapy

External beam therapy was given to patients with high-risk tumors and to many with medium-risk tumors. The target volume was the previous site of the uterus and adnexa, the parametria, the proximal two-thirds of the vagina, and the lymphatic drainage regions along the iliac vessels up to the promontory. The superior field border was set at the L5-S1 disk. The total dose to be delivered to this volume was 46 Gy (median dose 46.0 Gy, range 6–50 Gy) and daily fractions of 1.8–2.0 Gy (Table 3).

### 2.6. Data Management

All data were collected in a computerized database at the Regional Oncology Center, Örebro, Sweden.

### 2.7. Statistical Analyses

In the statistical analyses, survival curves were generated using the Kaplan-Meier technique, and differences were tested with the log-rank test. The Pearson chi-square test was used for comparison of proportions and the independent *t*-test for comparing means of two groups. Multivariate analysis of prognostic factors was performed using the Cox proportional hazards model and logistic regression analysis. Best subset analysis was performed with multivariate technique to find the most important prognostic factors and to find the most powerful combination of these factors. All *P* values were based on two-sided tests, with *P* < 0.05 considered statistically significant. The Statistica software package (StatSoft, Inc., Tulsa, OK, USA, version 10, 2010) was used for the statistical analyses. 

## 3. Results

### 3.1. Recurrence Rate

The overall recurrence rate of the complete series was 519 out of 4,543 cases or 11.4%. Eighty-seven vaginal recurrences (1.9%) were diagnosed in the complete series. The regional pelvic (excluding vaginal recurrence) recurrence rate was 2.3% (103 cases), and the locoregional (vaginal or pelvic, or both) recurrence rate was 4.2% (190 cases). Of 190 locoregional recurrences, 87 (46%) occurred at the vaginal site. The 5-year actuarial locoregional relapse rate was 3.6%. Distant recurrences (outside the pelvic area) were noted in 329 cases (7.2%), and the 5-year actuarial relapse rate was 6.6%. The median time to relapse in the complete series was 19.7 months (range 1–248 months). In the complete series, 370 out of 519 recurrences (71%) occurred within 3 years and 445 recurrences (86%) within 5 years. The median age of all patients was 67.0 years (23–99 years), for those with recurrences was 68.4 years, and those without recurrences was 66.4 years.

### 3.2. Predictive Factors for Tumor Recurrences

At least 12 prognostic factors are described in endometrial carcinoma (Table 4). Some of them are also predictive factors for treatment outcome and tumor recurrences. Eight of these factors (age, FIGO stage, histology, FIGO grade, nuclear grade, DNA ploidy, myometrial infiltration, and p53 expression) were analyzed in this study with regard to the risk of tumor recurrence, both total rate and locoregional and distant recurrences. In a multivariate logistic regression analysis, three of these factors (FIGO grade, depth of myometrial infiltration, and DNA ploidy) were independent and statistically significant with regard to overall recurrence rate and distant recurrences (Table 5). The fourth most important predictive factor was the nuclear grade (best subset analysis). For locoregional recurrences no significant results were noted for these risk factors. In a model building analysis with best subset technique, the FIGO grade, depth of myometrial infiltration, and DNA ploidy gave the best predictive information with regard to the risk of tumor recurrences. Addition of further factors (age, histology, nuclear grade) only marginally increased the predictive value of the model. The single most important factor was the FIGO grade. Depth of myometrial infiltration was the second most important, and DNA ploidy (aneuploidy) the third factor. In this series 23.7% of the tumors with evaluable DNA status (*n* = 1,613) were nondiploid (aneuploid).

### 3.3. Survival Analyses

At the last followup (March 2010), the number of patients alive was 2,764 (61%), dead of disease 819 (18%), and dead of intercurrent disease 960 (21%). Death from intercurrent disease was more common than death from the cancer disease. The five-year actuarial overall survival rate was 73% and the cancer-specific survival rate was 83%. Five-year overall survival after any relapse was 30%. The salvage rate was 44% (38/87) after isolated vaginal recurrences, 20% (21/103) after pelvic recurrences, and 6% (19/329) after distant recurrences. 

### 3.4. Prognostic Factors for Survival

Eight prognostic factors were analyzed with Cox proportional multivariate regression analyses and with overall and cancer-specific survival rate as the dependent variable. Seven of these factors were independent and statistically highly significant (Table 6). P53 expression, analyzed with immunohistochemistry, was the only nonsignificant factor. Tumor stage (stages III-IV versus I-II) was the single most important factor with a risk ratio of 4.2 (95% CI 3.5–5.0) for advanced tumor stage. Tumor grade (grade 3 versus 1-2) was the second most important prognostic factor with risk ratio 2.5 (95% CI 2.1–3.0). Depth of myometrial infiltration had the lowest risk ratio 1.3 (95% CI 1.1–1.6) among the seven significant risk factors. The nuclear grade of the tumor was significant and independent of the FIGO grade in multivariate analysis. DNA ploidy (aneuploid versus diploid) was also an important and significant prognostic factor with a risk ratio of 1.6 (95% CI 1.3–2.0) with regard to cancer-specific survival rate.

### 3.5. Risk Group Definitions

**Figure 1:**
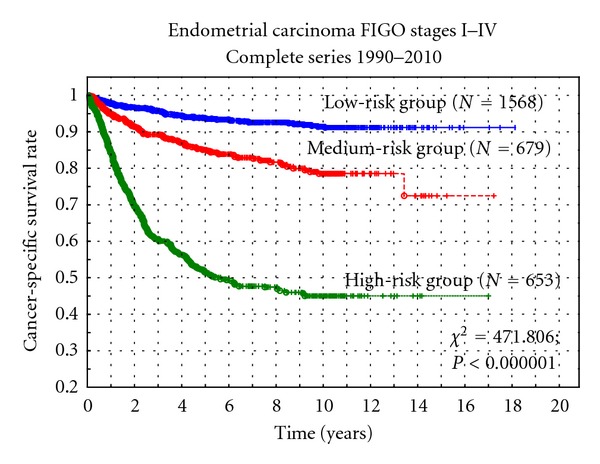
Cancer-specific survival rate versus three postoperative risk groups.

The risk group definitions presented under Material and Methods were used in the complete series and for all stages together and for stage I alone. In the complete series, 54% of the cases fulfilled low-risk criteria, 23% medium-risk criteria, and 22% high-risk criteria. In stage I, the corresponding figures were 57%, 25%, and 17%, respectively. The discriminating power (chi-square = 471.8; *P* < 0.000001) with regard to cancer-specific survival rate was very high both for the complete series and for stage I tumors alone. The 5-year survival rate in the high-risk group was only 50% in the complete series, and this group was very distinctly separated from the low-risk and medium-risk groups (Figure 1). On the other hand, the difference in survival between the last two groups was only 10% at 5 years.

**Figure 2:**
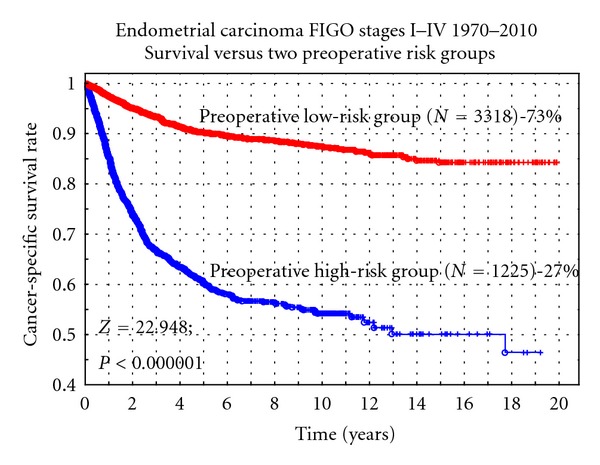
Cancer-specific survival rate versus two preoperative risk groups.

If instead only two risk groups are used, which are proposed for preoperative risk group definitions, a 30% difference was noted in cancer-specific survival at 5 years, which was also highly statistically significant (Z = 22.948; *P* < 0.000001). In FIGO stage I, the difference between the two groups was 20% (75% versus 95%) (Z = 12.980; *P* < 0.000001) (Figure 2). In the preoperative definitions, only three prognostic factors were used: histology (nonendometrioid versus endometrioid), FIGO grade (grade 3 versus 1-2), and DNA ploidy (nondiploid versus diploid). It was not necessary to use myometrial infiltration, which is not a reliable prognostic factor assessed preoperatively.

## 4. Discussion

The optimal treatment of endometrial carcinoma patients have been vividly discussed and also studied in a number of randomized trials during the last decades [1–9]. Before that, no consensus existed with regard to type of therapy, but the situation has changed, and our evidence-based knowledge in this field has improved substantially. However, still different conclusions are drawn from the available study data, and the optimal treatment of the various risk groups is continuously debated in various countries and in different centers. Various definitions of the risk groups have confused the results, and conclusions drawn from the studies [2–5, 7–9]. The PORTEC-1 study [2] included both low-risk (grade 2, superficial infiltration) and medium-risk cases, and the ASTEC/EN.5 study [4] included both medium-risk and high-risk cases. Most authors agree that low-risk cases can be left with surgery alone [7, 10, 11]. Still, vaginal brachytherapy is effective and will reduce the rate of vaginal recurrences in all risk groups, but from different levels [7]. From a cost-effectiveness perspective, it seems reasonable to exclude the low-risk tumors from this type of adjuvant therapy. Treatment of isolated vaginal relapse after surgery alone is effective in 89% (complete remission) with 65% survival rate [12]. In the present study, the salvage rate was only 44% after vaginal recurrences. For medium-risk cases, the situation is not clear. A number of studies have focused on this risk group, but still with various definitions of this risk group (medium-risk, low-medium risk, and high-medium risk) [1–9]. A mixture of both low-risk and medium-risk cases has been studied as well as a mixture of medium-risk and high-risk cases. Improved locoregional tumor control has been shown but so far no influence on survival [1–8]. From our country a randomized study has presented data for a pure medium-risk group that did include neither low-risk cases nor high-risk cases [9]. The low-risk and high-risk groups have been studied separately in two other randomized protocols, and the results have been presented elsewhere [7, 8]. 

The aim of the present study was to evaluate the prognostic value of the various clinical and histopathological factors commonly discussed in endometrial cancer and how to combine them into risk group definitions. Proposals of pre- and postoperative risk groups are presented and tested in a large series of endometrial carcinomas comprising more than 4,500 patients. The only large study published before analyzing prognostic factors in endometrial cancer was the registry SEER study, where 41,120 cases were included. FIGO stage, type of histology, FIGO grade, lymph node status, age at diagnosis, and race were found to be prognostic factors in that study [13]. 

The 5-year actuarial locoregional recurrence rate was 3.6%, and the distant recurrence rate was 6.6% in our series. Eight of twelve commonly used prognostic and predictive factors were analyzed in this study. With regard to recurrences FIGO grade, DNA ploidy, and depth of myometrial infiltration were independent and significant predictive factors in a multivariate logistic regression analysis. A best subset analysis also confirmed that these three factors were the most important ones, and addition of further factors only marginally improved the predictive value of the model. The single most important factor was the FIGO grade, but it is important to point out that the DNA ploidy, not so commonly used in the international literature, was one of the three most important predictive factors together with myometrial invasion to predict the risk of tumor recurrences, and especially distant recurrences. A number of studies from Sweden [14–17] have pointed out the prognostic importance of DNA ploidy before, but this information does not seem to have been generally accepted and spread worldwide [2–5].

The 5-year actuarial overall survival rate in this series of patients was 73%, and the cancer-specific survival rate was 83%. The study covers a long time period, but in fact the overall survival did not change during the last three decades. Changes in the treatment technique during these years seem to have had no impact on survival. Cox proportional multivariate regression analysis was used to find out the most important prognostic factors with regard to the cancer-specific survival probability. Eight factors were included in the model, and seven were found to be independent and significant. Of the included factors only p53 expression was nonsignificant in these analyses. Advanced versus early tumor stage was the single most important factor with a risk ratio of 4.2, and FIGO grade was the second most important with a risk ratio of 2.5. Interesting findings were that the nuclear grade [14, 15] was significant and independent of the FIGO grade, and the DNA ploidy with a risk ratio of 1.6 was more important than myometrial invasion with a risk ratio of 1.3. In fact, myometrial invasion had the lowest risk ratio of all seven analyzed and significant prognostic factors.

Tumor size and lymphovascular space invasion (LVSI) were not included in the present analyses, since these variables were not regularly reported by the departments of pathology during the extensive study period. Tumor size with a cutoff level of 2 cm has been reported to be an important predictive factor in preoperative risk classification to define a low-risk group where lymph node dissection is not required [18]. In another study tumor size was not an independent predictor of recurrence [19]. In a number of studies, LVSI has been pointed out as an important predictive factor associated with lymph node metastases and distant tumor spread [20–22].

Three risk groups were analyzed with the definitions used in our country during the last 20 years and also in three published randomized multicenter studies [7–9]. Interesting to note is that 22% of all tumors belonged to a high-risk group with these definitions and 54% belonged to a low-risk group. The prognoses of the three groups are highly significantly different, and especially the high-risk group showed a poor prognosis with only 50% cancer-specific survival rate. These definitions and risk groups seemed to work out well to discriminate between patients, where surgery alone is enough (low-risk cases), where vaginal brachytherapy should be added (medium-risk cases), and where external beam radiotherapy and chemotherapy probably are the treatment options [7–9].

For preoperative risk group classification it is more convenient to use two risk groups. The aim of this classification is to sort out those patients requiring lymph node dissection from those who do not. Myometrial invasion is an important predictive and prognostic factor but difficult to assess preoperatively in a reliable way. Our multivariate analyses of this large series of patients have shown that myometrial invasion can be replaced by other prognostic factors without losing to much of prognostic information. The results from our analysis showed that histology (nonendometrioid versus endometrioid), FIGO grade (grade 3 versus grade 1-2), and DNA ploidy (nondiploid versus diploid) could be used to define two preoperative risk groups. These two risk groups discriminated well (*P* < 0.000001) between low-risk and high-risk cases with a 30% difference in 5-year cancer-specific survival rate. Using this definition, the preoperative high-risk group includes 27% of all new cases of endometrial cancer. In an Italian study, preoperative risk classification was made using histology, tumor grade, myometrial invasion, cervical spread, and abdominal spread and correctly identified the postoperative risk classification in 96% with high sensitivity and specificity [23]. This may help the surgeon in the decision to perform limited or extended surgery.

The importance of DNA ploidy as a predictive and prognostic factor in endometrial carcinoma [14–17, 24] and part of risk group classifications [7–9] is one of the most important results of this study. It is important to analyze large samples of endometrial carcinomas to sort out the most important and significant predictive and prognostic factors that should be used in future risk group classifications. It is also important for coming randomized studies that there will be an international consensus regarding the definition criteria to be used for the various risk groups. 

## 5. Conclusions 

Risk group definitions are important in the design of randomized studies in endometrial carcinomas. Up to now some confusion exists in these definitions in published randomized studies making firm conclusions and comparisons difficult. Three risk groups seem reasonable to use in the postoperative setting, but probably only two in the preoperative classification. Our study has shown that DNA ploidy is an important predictive and prognostic factor and if used in combination with the FIGO grade and type of histopathology can replace myometrial invasion in definition of preoperative high-risk cases needing more extensive surgery. 

## Figures and Tables

**Table:** (a) FIGO stage (clinical)

IA	1 388
IB	865
II	185
III	108
IV	97
None	1 900

Total	4 543

1 840 patients were staged both clinically and surgically.

**Table:** (b) FIGO stage (surgical)

I*	899
IA	91
IB	1 453
IC	625
II*	130
IIA	50
IIB	100
III*	82
IIIA	84
IIIB	11
IIIC	68
IVA	3
IVB	144
None	803

Total	4543

*No further substage.

**Table 2:** Tumor characteristics of the complete series.

Histology		
Endometrioid	3 971	87.4%
Nonendometrioid	323	7.1%
Unspecified	249	5.5%

Nuclear grade		
1	538	11.8%
2	746	16.4%
3	433	9.5%
Unknown	2 866	62.2%

P53 status		
Positive	249	5.5%
Negative	258	5.7%
Unknown	4 036	88.8%

Myometrial infiltration		
Endometrium alone	95	2.1%
≤50%	1 562	34.4%
>50%	848	18.7%
Unknown	2 038	44.9%

FIGO grade		
1	1 433	31.5%
2	1 808	39.8%
3	850	18.7%
Unknown	452	9.9%

DNA ploidy		
Diploid	1 231	27.1%
Nondiploid	382	8.4%
Unknown	2 930	64.5%

**Table 3:** Techniques used for the external beam pelvic radiotherapy.

Parameter	Specification
Type of field	4-field box technique
Radiation quality	6–50 MV (linear accelerators)
Dose per fraction	1.8–2.0 Gy
Number of fractions	23 (median) (range 5–26)
Total dose	46.0 Gy (median) (range 6–50 Gy)
Fractionation	Daily fractions, 5 fractions per week
Superior field border	L5-S1 disk
Inferior field border	Lower margin of the fossa obturatoria
Lateral borders	1 cm lateral of the linea terminals

**Table 4:** Prognostic factors in endometrial carcinoma.

(1) FIGO stage (clinical, surgical)*	
(2) Tumor size (>2 cm)	
(3) Histology (endometrioid, nonendometrioid)*	
(4) Myometrial infiltration (>50%)*	
(5) FIGO grade (grade 3 versus 1-2)*	
(6) Nuclear grade (grades 1–3)*	
(7) DNA ploidy (diploid, nondiploid)*	
(8) S-phase fraction	
(9) P53 expression (positive versus negative)*	
(10) ER and PgR expression	
(11) Lymphovascular space invasion (LVSI)	
(12) Age of the patient (>60 years)*	

^∗^Factors analysed in the present study.

**Table 5:** Multivariate logistic regression analyses of factors predicting recurrences.

Factor	Odds ratio	95% CI	*P* value
Overall tumor recurrences			
Age (>60 years)	1.300	0.619–2.729	0.489
Histology*	1.092	0.556–2.147	0.798
FIGO grade (3 versus 1-2)	3.726	1.957–7.095	0.00006
Nuclear grade (3 versus 1-2)	1.713	0.878–3.341	0.114
DNA ploidy**	1.669	1.071–2.602	0.024
Myometrial infiltration***	2.077	1.392–3.098	0.0003

Distant tumor recurrences			
Age (>60 years)	0.844	0.377–1.889	0.680
Histology*	1.155	0.572–2.330	0.689
FIGO grade (3 versus 1-2)	4.750	2.319–9.729	0.00002
Nuclear grade (3 versus 1-2)	1.686	0.809–3.515	0.163
DNA ploidy**	1.805	1.101–2.957	0.019
Myometrial infiltration***	2.853	1.784–4.565	0.00001

^∗^Nonendometrioid versus endometrioid. **Nondiploid versus diploid. ***>50% versus <50%.

**Table 6:** Multivariate Cox proportional regression analyses of prognostic factors.

Factor	Risk ratio	95% CI	*P* value
Overall survival rate			
Age (>60 years)	2.951	2.467–3.531	<0.00001
FIGO stage (III-IV versus I-II)	2.489	2.169–2.855	<0.00001
Histology*	1.569	1.340–1.836	<0.00001
FIGO grade (3 versus 1-2)	1.814	1.590–2.069	<0.00001
Nuclear grade (3 versus 1-2)	1.558	1.286–1.888	<0.00001
DNA ploidy**	1.456	1.215–1.744	<0.0001
Myometrial infiltration***	1.322	1.164–1.501	<0.0001
P53 expression****	0.955	0.715–1.276	0.755

Cancer-specific survival rate			
Age (>60 years)	1.925	1.509–2.456	<0.00001
FIGO stage (III-IV versus I-II)	4.205	3.542–4.993	<0.00001
Histology*	1.717	1.410–2.090	<0.00001
FIGO grade (3 versus 1-2)	2.524	2.099–3.035	<0.00001
Nuclear grade (3 versus 1-2)	1.635	1.279–2.090	<0.0001
DNA ploidy**	1.591	1.267–1.999	<0.0001
Myometrial infiltration***	1.292	1.069–1.561	0.008
P53 expression****	1.089	0.747–1.587	0.657

^∗^Nonendometrioid versus endometrioid. **Nondiploid versus diploid. ***>50% versus <50%. ****Positive (>30% staining) versus negative (<30% staining).
